# The SIRT1/STAT3 axis as a central regulator of immune, inflammatory, and lipid metabolic dysregulation in rheumatoid arthritis: therapeutic implications

**DOI:** 10.3389/fimmu.2026.1746043

**Published:** 2026-06-02

**Authors:** Chengzhi Cong, Yuan Wang, Jian Liu

**Affiliations:** 1 Department of Rheumatology and Immunology, First Affiliated Hospital of Anhui University of Chinese Medicine, Hefei, Anhui, China; 2 First Clinical Medical School, Anhui University of Chinese Medicine, Hefei, Anhui, China; 3 Anhui Provincial Key Laboratory for Applied Basic and Clinical Translational Research on Rheumatologic Diseases in Traditional Chinese Medicine, Anhui Academy of Chinese Medicine, Hefei, Anhui, China

**Keywords:** immunometabolism, lipid dysregulation, rheumatoid arthritis, SIRT1/STAT3, therapeutic target

## Abstract

Rheumatoid arthritis (RA) is a chronic autoimmune disease characterized by synovial inflammation, joint destruction, and systemic comorbidities, particularly cardiovascular disease (CVD) associated with severe dyslipidemia. The bidirectional crosstalk between immune-inflammatory processes and lipid metabolic disturbances is increasingly recognized as a key driver of RA pathogenesis, yet the molecular mechanisms integrating these domains remain poorly understood. This review synthesizes current evidence to propose the Sirtuin 1 (SIRT1)/signal transducer and activator of transcription 3 (STAT3) axis as a central regulator of immune, inflammatory, and lipid metabolic dysregulation in RA. SIRT1, an NAD^+^-dependent deacetylase, functions as a metabolic sensor with anti-inflammatory and lipid-regulating properties, whereas STAT3 acts as a proinflammatory transcription factor driving Th17 differentiation, synovial hyperplasia, and metabolic reprogramming. SIRT1 directly deacetylates and inactivates STAT3, establishing an antagonistic yin–yang relationship. In RA, chronic inflammation and metabolic stress suppress SIRT1 and hyperactivate STAT3, creating a positive feedback loop that perpetuates immune imbalance and lipid dysfunction. We further discuss therapeutic strategies targeting this axis, including SIRT1 activators, STAT3 inhibitors, and dietary interventions such as *n*-3 polyunsaturated fatty acids, which hold promise for simultaneously mitigating inflammation and correcting metabolic abnormalities in RA. This integrated perspective challenges the traditional siloed approach and opens new avenues for immunometabolic therapy in RA.

## 1 Introduction

Rheumatoid arthritis (RA) is a chronic systemic autoimmune disease caused by persistent synovial inflammation, progressive cartilage and bone destruction, and various extra-articular manifestations (1, 2). Affecting up to 0.5%–1% of the world’s population, RA imposes a significant burden on patients and healthcare systems alike, owing to chronic pain, disability related to impaired function, and accelerated comorbid conditions—most notably cardiovascular diseases (CVD) (3, 4). The etiology of RA reflects a combination of genetic susceptibility, altered innate and adaptive immunity responses, and environmental triggers (5, 6). Traditionally, studies and treatments focused on controlling the inflammatory process by blocking the production of cytokines—such as tumor necrosis factor alpha (TNF-α), and interleukins (e.g., IL-6)—with excellent clinical response (7, 8). However, a large proportion of patients display poor responses, and the specific factors responsible for the chronic, systemic nature of RA remain poorly understood.

In addition to the established inflammatory model, accumulating evidence highlights the crucial involvement of widespread metabolic derangements in the RA etiology (9, 10). Of particular relevance is the prevalence of dyslipidemia in RA, characterized by the so-called lipid paradox: despite lower total cholesterol and low-density lipoprotein cholesterol (LDL-C), RA patients remain at markedly increased risk of CVD (11, 12). This paradox suggests that qualitative and functional changes in lipoproteins and lipid mediators, rather than quantitative alterations, drive the pathology. Lipidomic analyses have demonstrated alterations in lipid composition within the plasma and synovial fluid of RA patients, favoring proinflammatory lipid species (e.g., prostaglandins and leukotrienes derived from arachidonic acid) over proresolving lipid mediators (e.g., resolvins and maresins derived from *n*-3 polyunsaturated fatty acids) (13, 14). These lipid mediators are neither spectators nor passive agents in the immune response; rather, they actively modulate the activation, differentiation, and effector functions of T cells, B cells, macrophages, and fibroblast-like synoviocytes (FLS) (15, 16). Such complex interaction establishes a vicious circle: inflammation stimulates lipid metabolism disorder, which in turn stimulates inflammation and sustains it (17).

Although we have identified this “immune-inflammatory–lipid metabolic disorder” network, it is crucial to delineate the key molecular machineries that link pathological signals from both inflammatory stimuli and metabolic stress to orchestrate disease consequences in RA. Which molecular regulator senses inflammatory cues and metabolic stress to trigger pathological outcomes in/ RA? We propose that this axis consists of the nicotinamide adenine dinucleotide (NAD^+^)-dependent deacetylase Sirtuin 1 (SIRT1) and the signal transducer and activator of transcription 3 (STAT3).

SIRT1 is a mediator of metabolic and stress-regulatory responses, with the potential to boost oxidative metabolism, modulate mitochondrial metabolic flux, and exert substantial anti-inflammatory effects (18, 19). Meanwhile, STAT3 functions as the chief transcription factor in response to cytokines and growth factors, governing cell division, survival, and proinflammatory gene expression (20, 21). Despite not being entirely independent, these two pathways form an intimate connection through a direct and antagonistic relationship. SIRT1 deacetylates and thereby deactivates STAT3, functioning as a molecular brake on its transcriptional activity (22, 23). Under RA conditions, chronic inflammatory signaling and metabolic stress (i.e., raised free fatty acids, oxidative stress) are known to dampen SIRT1 activity while simultaneously activating STAT3 (24, 25). Collectively, this disequilibrium threatens immune homeostasis, fosters a catabolic synovial milieu, and disrupts systemic lipid metabolism.

In this review, we set out to summarize current evidence supporting a new paradigm: the SIRT1/STAT3 pathway as a key regulatory hub of RA immunometabolism. We will examine how SIRT1 and STAT3 contribute to both immune dysregulation and lipid metabolism disorder in RA and explain how their imbalance forms a self-reinforcing feedback loop. Moreover, we will analyze the therapeutic promise of targeting this axis, suggesting that interventions capable of modulating the SIRT1/STAT3 axis could provide a unified approach in the effort to simultaneously dampen inflammation and correct metabolic perturbations in RA, thereby improving disease control and reducing comorbidities.

## 2 Immune-inflammation and lipid metabolic dysregulation in RA: an intertwined pathological circuit

### 2.1 Immune dysregulation

RA synovium constitutes a coordinated site of immune pathology resulting from adaptive changes in the selected cell populations. Tolerance is breached, paving the way for activation of self-reactive T cells (T helper 17 [TH17]). Cytokines IL-6, IL-1β, and IL-23 precisely drive the differentiation of these cells through activation of the transcription factor STAT3 and consequent synthesis of IL-17 and IL-22. These cytokines subsequently promote synovial fibroblasts and macrophages to upregulate expression of additional inflammatory cytokines and matrix-degrading enzymes, forming an upregulating cycle (26, 27). Simultaneously, metabolism of inflammatory pathways—particularly the immune-regulatory role of the AMP-activated protein kinase (AMPK) pathway—and the functions and quantity of regulatory T (Treg) cells, which are essential for maintaining immune homeostasis, are hindered (28). This Th17/Treg skewing imbalance serves as a key rheostat in RA autoimmunity. Myeloid cells also promote inflammation: macrophages polarized towards an M1 phenotype through IFN-γ and Toll-like receptor (TLR) ligands release large amounts of TNF-α, IL-1β, and IL-6, while synovial neutrophils release neutrophil extracellular traps (NETs) that strengthen antigen presentation and complement activation (29–31). Importantly, resident FLS undergo pathogenic imprinting, becoming a “signature of imprinted aggressiveness” characterized by epigenetic marks, cell-apoptotic resistance, and migration and invasion of the cartilage beyond their interactions with immune cells (32). This complex immune dysregulation is sustained by direct perturbation of systemic metabolism by a persistent inflammatory signal.

### 2.2 Lipid metabolism disorders

The RA lipid profile exhibits multiple inflammation-mediated disturbances that extend far beyond simple changes in concentration and manifest pathologically. The “lipid paradox” is well documented by the finding that active RA is characterized by low circulating total cholesterol (TC) and LDL-C, and yet carries a high risk of CVD (33, 34). The antioxidant and anti-inflammatory properties of high-density lipoprotein (HDL) are largely depleted. This abnormal behavior is caused by the substitution of protective enzymes, such as paraoxonase-1 (PON1), with acute-phase proteins, such as serum amyloid A (SAA), and by oxidative changes related to the oxidative and proteolytic activity of myeloperoxidase (MPO) in the inflamed joint and vasculature (35, 36). The LDL pool begins to favor an abundance of small, dense LDL particles that are more prone to oxidation and more readily penetrate the vasculature. Oxidized low-density lipoprotein (ox-LDL) not only promotes strong atherogenicity but also acts as a damage-associated molecular pattern (DAMP), stimulating pattern-recognition receptors expressed on immune cells to promote inflammation (37, 38). At the lipid bioactive level, proinflammatory versus proresolving mediators are in gross imbalance. The synovial fluid is a rich source of eicosanoids, such as prostaglandin E2 (PGE2), which causes vasodilation and pain, and leukotriene B4 (LTB4), a powerful neutrophil chemoattractant (39, 40), derived from arachidonic acid (AA). In contrast, factors that should function as prosolving mediators (SPMs)—such as resolvins [from eicosapentaenoic acid (EPA) and docosahexaenoic acid (DHA)] and lipoxins (from AA)—are commonly defective, resulting in a failure of the natural resolution of inflammation (41). The disruption of resolution pathways is as important as the primary inflammatory burst in sustaining chronic disease.

### 2.3 Mechanistic crosstalk: linking lipid pathways to inflammatory signaling

**Figure 1:**
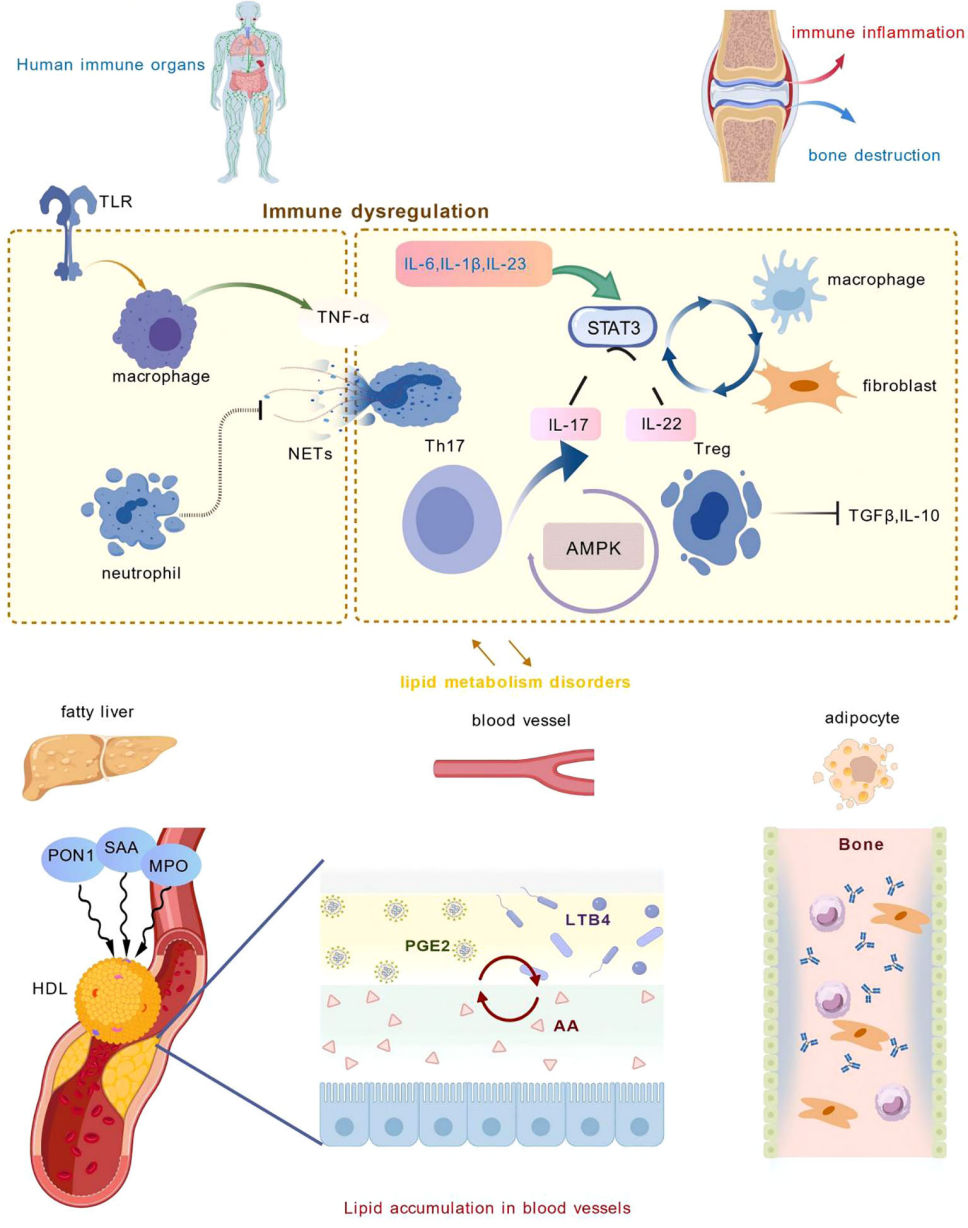
The intertwined pathological circuit of immune dysregulation and lipid metabolism disorders in rheumatoid arthritis. This schematic illustrates the self-reinforcing cycle between immune inflammation and lipid dysregulation in RA. In the inflamed synovium, IL-6 drives STAT3-dependent Th17 differentiation, while Treg function is suppressed. Systemic inflammation promotes dysfunctional HDL (loss of PON1, gain of SAA/MPO) and shifts the lipid mediator balance toward proinflammatory eicosanoids (PGE2, LTB4). Black arrows denote reciprocal positive feedback: cytokines alter lipid metabolism (e.g., by promoting atherogenic sdLDL), while proinflammatory lipids and free fatty acids amplify immune activation (e.g., via TLR4), thereby perpetuating disease chronicity. Created with BioGDP.com (128).

This crosstalk is mechanistic and reciprocal, feeding into a vicious cycle. Certain lipid species directly modulate immune cell function through defined signaling pathways. Saturated free fatty acids (e.g., palmitate) stimulate the Toll-like receptor 4 (TLR4)/nuclear factor kappa-light-chain-enhancer of activated B cells (NF-κB) axis in macrophages and FLS, enhancing IL-6 and TNF-α production (42). In contrast, *n*-3 polyunsaturated fatty acids (PUFAs), such as DHA, are anti-inflammatory not only as precursors to SPMs but also by suppressing the NACHT, LRR, and PYD domain-containing protein 3 (NLRP3) inflammasome and activating the anti-inflammatory transcription factor nuclear factor erythroid 2-related factor 2 (NRF2) (43, 44). Intracellular cholesterol accumulation within immune cells alters membrane fluidity and lipid raft composition, thereby affecting the receptor organization and signaling—such as the T-cell receptor (TCR) and the B-cell receptor (BCR)—in regulating the cell activation threshold (45). Inflammatory cytokines, meanwhile, act systemically to rewire lipid metabolism: TNF-α and IL-1β suppress the expression of reverse cholesterol transport proteins such as ATP-binding cassette transporter A1 (ABCA1) in the macrophages, favoring foam cell formation. They also inhibit lipoprotein lipase (LPL) function, leading to hypertriglyceridemia and increased hepatic *de novo* lipogenesis, further altering the plasma lipidome (46, 47). This establishes a pathological feed-forward loop: inflammation alters lipid metabolism to generate proinflammatory lipid species that, in turn, amplify the inflammatory response, driving disease progression and comorbidities such as atherosclerosis. **Figure 1** illustrates the pathological circuit in which immune inflammation and lipid metabolism disorders interweave in RA.

## 3 The SIRT1/STAT3 axis: an integrative hub at the crossroads of immunity and metabolism

### 3.1 SIRT1: a metabolic sensor and epigenetic regulator of cellular homeostasis

SIRT1, a member of the class III histone deacetylases, functions as a key sensor of cellular metabolic status, translating metabolic flux directly into transcriptional programs through its NAD^+^ dependency (48). Importantly, this activity extends beyond chromatin remodeling: SIRT1 acts with high fidelity on the nonhistone targets to serve as a major gatekeeper to metabolic homeostasis. By deacetylating and activating peroxisome proliferator-activated receptor gamma coactivator 1-alpha (PGC-1α), SIRT1 promotes mitochondrial biogenesis and fatty acid β-oxidation, thereby enforcing an oxidative metabolic phenotype that is often suppressed in hyperactive immune cells (49, 50). SIRT1 also regulates lipid metabolism by stimulating cholesterol efflux through activation of liver X receptors (LXRs) and repressing the activity of sterol regulatory element-binding proteins (SREBPs), the central transcriptional regulators of lipogenesis (51, 52). In addition, SIRT1 exerts potent anti-inflammatory effects by deacetylating the RelA/p65 subunit of NF-κB, thereby inhibiting transcription of proinflammatory cytokines (e.g., TNF-α and IL-6) (53, 54). In RA synovial tissues and in animal models of arthritis, SIRT1 activity is often reported to be downregulated, likely due to local factors such as NAD^+^ depletion and oxidative stress (55, 56). However, systemic levels of SIRT1 in RA patients appear more complex and context-dependent, with some clinical studies suggesting compensatory upregulation—an unresolved area of ongoing research (57).

### 3.2 STAT3: a signaling nexus for inflammation, survival, and metabolic reprogramming

STAT3 is a pleiotropic transcription factor that converges on signaling from a wide variety of cytokines, principally those of the IL-6 family (58, 59). In RA, sustained inflammatory signaling activates STAT3, which is phosphorylated by JAKs, dimerizes, and translocates to the nucleus to modulate a broad genetic program (60, 61). STAT3 is essential for the differentiation and pathogenicity of Th17 cells, directly driving transcription of genes encoding IL-17 and the IL-23 receptor (62, 63). Confirmed as a key therapeutic target in RA, STAT3 plays a central role in sustaining the pathogenic Th17 response (64). STAT3 also induces cell survival and hyperproliferation by upregulation of antiapoptotic molecules such as Bcl-xL, which directly participate in synovial hyperplasia and resistance of FLS to apoptosis (65). Beyond these two “canonical” roles, STAT3 has also been implicated in metabolic reprogramming because of its capacity to favor glycolytic flux (Warburg effect) to satisfy the biosynthetic needs of proliferating inflammatory cells (66, 67). The transcriptional potential of STAT3 is not exclusively controlled by phosphorylation; acetylation, particularly at lysine residue K685, considerably enhances its DNA-binding affinity and stability, thereby adding another crucial layer of posttranscriptional regulation (68, 69).

### 3.3 Mechanistic integration: SIRT1 as a direct brake on STAT3-driven pathology

**Figure 2:**
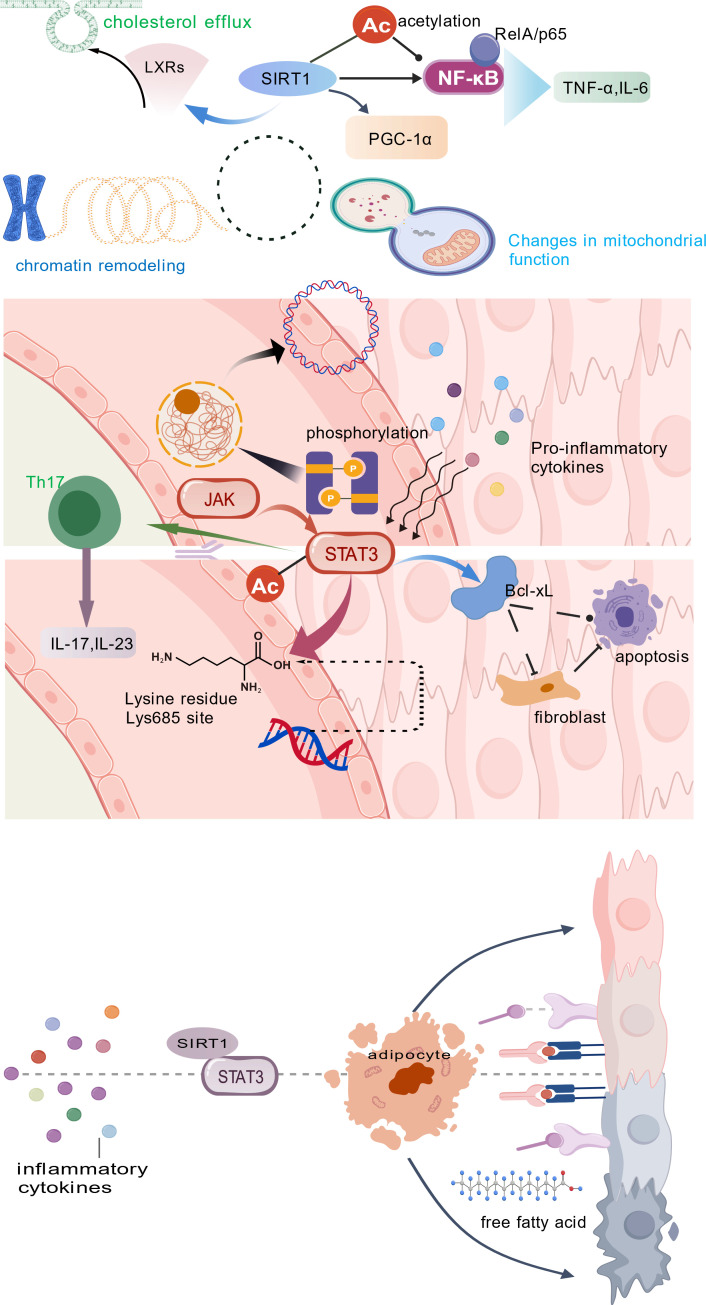
The SIRT1/STAT3 Axis as an Integrative Hub at the Intersection of Immunity and Metabolism. This diagram depicts the antagonistic relationship between SIRT1 and STAT3 and its immunometabolic consequences. SIRT1-mediated homeostasis: SIRT1 deacetylates PGC-1α (mitochondrial biogenesis) and NF-κB (anti-inflammatory), and activates LXR to promote cholesterol efflux. STAT3-driven inflammation: cytokines (e.g., IL-6) activate STAT3 via JAK phosphorylation; acetylation at Lys685 enhances its transcriptional activity, thereby driving Th17 differentiation (IL-17, IL-23) and cell survival (Bcl-xL). Central antagonism: SIRT1 directly deacetylates STAT3 at Lys685, acting as a molecular brake on its proinflammatory program. In RA, inflammatory and metabolic stressors suppress SIRT1 and hyperactivate STAT3, thereby creating a pathogenic feed-forward loop. Created with BioGDP.com (128).

The SIRT1/STAT3 axis consists of their direct molecular interaction, conferring to the SIRT1/STAT3 axis a functionally imperative, antagonistic relationship. SIRT1 is a key negative regulator of STAT3 through a direct deacetylation of Lys685 (70, 71). SIRT1-mediated deacetylation of STAT3 acts as a molecular brake that downregulates STAT3 activity in terms of its DNA-binding propensity and ultimate transcription of proinflammatory/prosurvival/prometabolic genes (72, 73), forming a yin–yang axis linking cellular energy status, monitored through NAD^+^ and SIRT1, to regulation of inflammatory signal amplitude. The second challenge, within this pathological state of RA, is that the combination of inflammatory cytokines with metabolic stress (for example, increased free fatty acids) constitutes the ideal combination: SIRT1 is shut off, while cytokine signaling hyperactivates STAT3 (74, 75). Beyond directly deacetylating STAT3, SIRT1 also indirectly modulates STAT3-driven pathology. For instance, by activating PGC-1α, SIRT1 promotes fatty acid oxidation—a metabolic program essential for Treg cells—while simultaneously suppressing the NF-κB pathway, thereby reducing the overall inflammatory milieu that sustains STAT3 activation (49, 54). The SIRT1/STAT3 axis is therefore not two parallel circuits but an integrated core loop, whose dysfunction represents one of the central mechanisms driving the immunometabolic dysfunction of RA. **Figure 2** illustrates the mechanism of the SIRT1/STAT3 axis as an integrated hub at the intersection of immunity and metabolism.

### 3.4 A synthesis of the SIRT1/STAT3 axis: established mechanisms, emerging hypotheses, and pathophysiological linkages

The SIRT1/STAT3 axis integrates metabolic sensing with inflammatory signaling through several layers of regulation, the evidentiary support for which varies considerably. It is well established, based on direct biochemical and genetic evidence, that STAT3 is a master transcription factor for Th17 differentiation and proinflammatory gene expression (62–64), and that SIRT1 directly deacetylates STAT3 at Lys685, thereby inhibiting its transcriptional activity (70, 71). These findings are consistently supported by *in vitro* and *in vivo* models. The regulation of SIRT1 activity in RA, however, is complex: while downregulated in the synovium (55, 74), systemic levels may be upregulated, representing either a compensatory mechanism or a point of scientific debate (57).

In contrast, several aspects of this axis represent speculative or emerging hypotheses that require further validation. The precise quantitative relationship between the degree of hepatic STAT3 activation, the extent of SIRT1 suppression, and the resulting shift in the lipoprotein profile towards small, dense LDL (sdLDL) particles in RA patients remains to be fully established. Similarly, while SIRT1 activation enhances LXR-mediated cholesterol efflux *in vitro* (52, 76), the extent to which this pathway can be therapeutically exploited to reverse HDL dysfunction and mitigate the elevated atherogenic risk in RA is an active area of investigation. The concept that restoring the SIRT1/STAT3 balance can simultaneously resolve synovial inflammation and correct systemic dyslipidemia represents a promising, though still emerging, therapeutic paradigm.

The integration of these pathways establishes a direct mechanistic link from the inflamed synovium to the vascular wall. In brief, systemic inflammatory cytokines activate hepatic STAT3, promoting a proatherogenic lipoprotein profile. Concurrently, inflammation-driven suppression of SIRT1 impairs LXR-dependent cholesterol efflux pathways ATP-binding cassette subfamily G member 1 (ABCA1/G1), crippling reverse cholesterol transport and contributing to dysfunctional HDL (77, 78). This dual mechanism provides a molecular rationale for the lipid paradox and directly connects the SIRT1/STAT3 signaling node to the heightened cardiovascular risk observed in RA patients.

## 4 The SIRT1/STAT3 axis in RA immunoinflammation

### 4.1 Th17/Treg imbalance

The equilibrium between proinflammatory Th17 cells and anti-inflammatory Treg cells is directly determined by the SIRT1/STAT3 axis. The pro-RA cytokine milieu favors chronic activation of STAT3, a process confirmed as a key therapeutic target in RA (64). In the RA synovium, downregulated SIRT1 fails to restrain STAT3 activity, leading to hyperacetylated STAT3 that drives transcription of Th17-specific genes (e.g., RORγt, IL-17A) (79–81). In addition, SIRT1 deficiency exerts a direct influence on Treg biology. SIRT1 deacetylates and stimulates the Treg master transcription factor Foxp3, stabilizing its function and enhancing suppressive activity (82, 83). Critically, this immunoregulatory imbalance is reinforced by underlying metabolic programming: Th17 cells are highly glycolytic, relying on aerobic glycolysis to sustain proliferation and effector functions, whereas Treg cells primarily utilize fatty acid oxidation (FAO) for energy (84). By promoting PGC-1α-mediated FAO and suppressing the hyperglycolytic state through NF-κB inhibition, SIRT1 intrinsically favors Treg differentiation and function while curbing Th17 pathogenicity (85). Conversely, STAT3 activation drives the glycolytic reprogramming essential for Th17 cell fate, establishing a metabolic feedback loop that further exacerbates the Th17/Treg imbalance (86). Thus, the synergy between uncontrolled STAT3-mediated Th17 differentiation and impaired SIRT1-mediated Treg suppression culminates in severe polarization, resulting in sustained inflammatory/autoimmune responses.

### 4.2 Macrophage polarization and neutrophil activity

In myeloid cells, the SIRT1/STAT3 axis dictates polarization state and inflammatory output. Macrophages in the RA synovium are predominantly skewed toward the proinflammatory M1 phenotype. Although STAT3 signaling is complex, its activation contributes to M1 polarization by sustaining proinflammatory gene transcription and suppressing anti-inflammatory pathways (87, 88). SIRT1 normally promotes a shift towards the M2 phenotype by deacetylating and inhibiting NF-κB and activating AMPK signaling (89, 90). Thus, suppression of SIRT1 removes a critical brake on inflammation, and the low SIRT1/high STAT3 state locks macrophages in a proinflammatory M1 mode. In neutrophils, STAT3 activation promotes survival and drives the release of NETs, which expose autoantigens and amplify immune complex formation (91). The role of SIRT1 in neutrophils is less well defined but is implicated in limiting excessive inflammatory responses and NETosis (92). Dysregulation of this axis, therefore, contributes to the persistence and hyperactivity of myeloid cells within the synovium.

### 4.3 Activation and invasion of fibroblast-like synoviocytes

**Figure 3:**
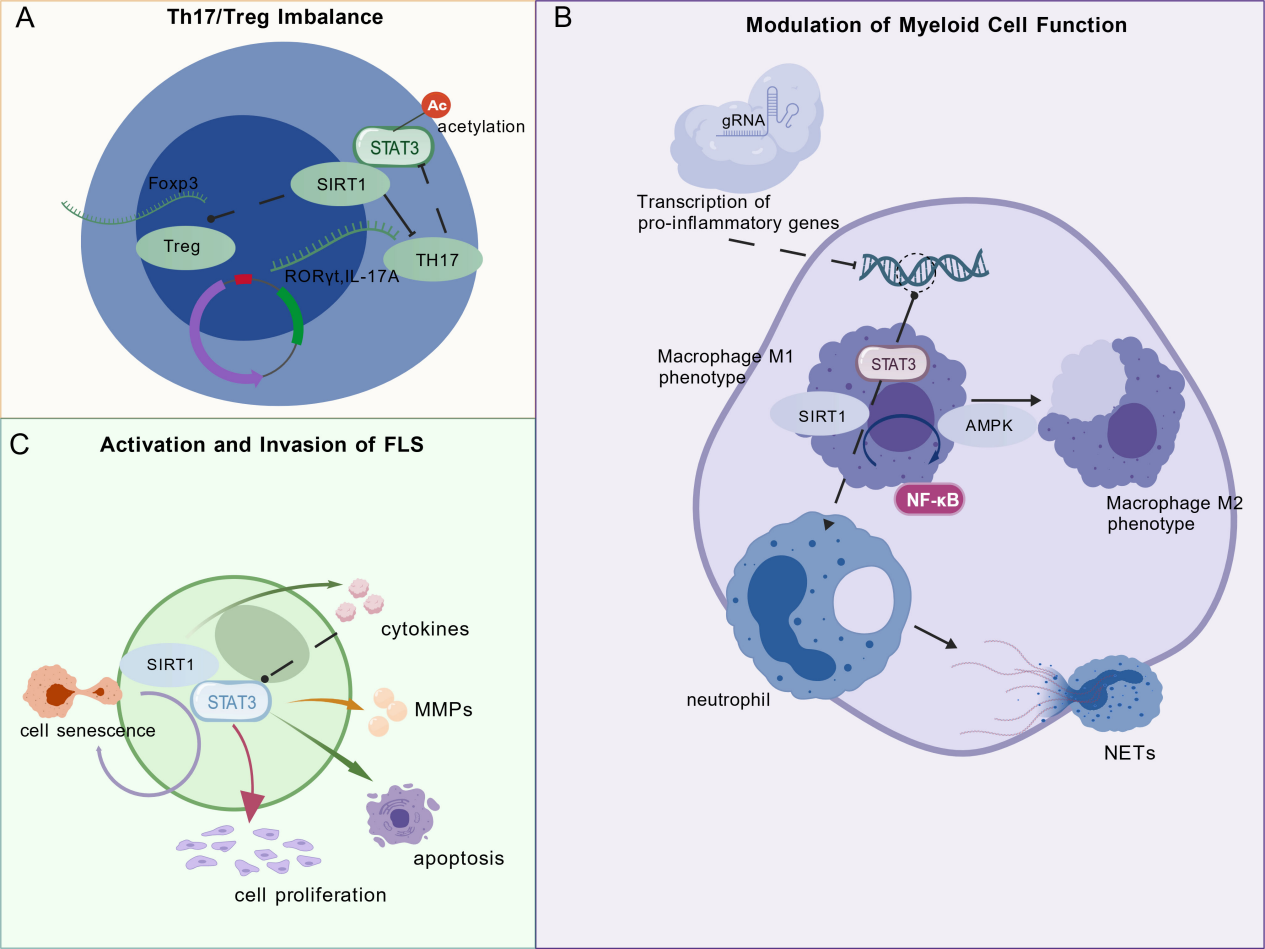
Cell type-specific consequences of SIRT1/STAT3 axis dysregulation in RA pathogenesis. SIRT1/STAT3 imbalance drives distinct pathogenic phenotypes in three key cell types. **(A)** Th17/Treg imbalance: low SIRT1 fails to deacetylate STAT3 and Foxp3, favoring STAT3-driven Th17 polarization over Treg stability. **(B)** Myeloid cell function: suppressed SIRT1 and activated STAT3 lock macrophages in a proinflammatory M1 phenotype and promote neutrophil NETosis. **(C)** Fibroblast-like synoviocytes (FLS): loss of SIRT1-mediated restraint and STAT3 hyperactivation drive FLS proliferation, apoptosis resistance, and MMP-mediated cartilage invasion. Created with BioGDP.com (128).

Activation of RA FLS into an aggressive, invading phenotype underlies joint destruction, with the SIRT1/STAT3axis as a key mediator. STAT3 activation, sustained by the cytokine milieu in the synovium, stimulates proliferation, apoptosis resistance, and production of matrix metalloproteinases (MMPs) and chemokines (93, 94). In contrast, SIRT1 functions as a tumor-suppressor-like molecule in FLS, whose activation induces apoptosis and inhibits invasive properties (95, 96). Pathological downregulation of SIRT1 in RA FLS—likely driven by oxidative and nitrosative stress—removes this restraint, thereby unleashing STAT3-mediated proliferative and invasive signaling. In addition, SIRT1 loss in FLS can result in premature senescence and SAS, which feeds back into the inflammatory environment through the secretion of proinflammatory molecules (97). This establishes a feed-forward loop in which inflammation-induced inhibition of SIRT1 enhances STAT3 activity and FLS activation, thereby driving further production of inflammatory mediators. **Figure 3** illustrates the mechanism of action of the SIRT1/STAT3 axis across different cell types in RA immune inflammation.

## 5 Governing lipid dysregulation in RA

### 5.1 Systemic regulation of lipoprotein metabolism

The lipid paradox in RA—characterized by seemingly normal or low circulating LDL-C levels in the face of elevated cardiovascular risk—can be mechanistically deconstructed through dysregulation of the SIRT1/STAT3 axis. The impact of this axis on systemic lipid homeostasis is multifaceted, operating through at least three coordinated mechanisms.

#### 5.1.1 STAT3-driven generation of proatherogenic lipoproteins

Systemic inflammation, particularly via IL-6, hyperactivates STAT3 in the liver. This hepatic STAT3 signaling does not necessarily increase the quantity of LDL particles but profoundly alters their quality. It promotes the generation of sdLDL particles, which are more susceptible to oxidation and penetrate the arterial wall more readily than larger, buoyant LDL particles, thereby enhancing their proatherosclerotic potential (98, 99).

#### 5.1.2 SIRT1 suppression impairs reverse cholesterol transport

Concurrently, inflammation-driven suppression of hepatic SIRT1 activity cripples a major atheroprotective pathway. Specifically, loss of SIRT1-mediated deacetylation impairs the activity of liver X receptor alpha (LXRα), a nuclear receptor that transcriptionally controls the ATP-binding cassette transporters ABCA1 and ATP-binding cassette subfamily G member 1 (ABCG1) (52, 76). These transporters are essential for mediating cholesterol efflux from peripheral tissues (e.g., macrophages in the artery wall) to nascent HDL particles, the critical first step in reverse cholesterol transport (RCT) (77). Therefore, SIRT1 deficiency results in impaired cholesterol clearance.

#### 5.1.3 Inflammation-induced HDL dysfunction

The combined effects of STAT3-driven sdLDL generation and SIRT1-suppressed RCT converge to promote a dysfunctional HDL phenotype. Under inflammatory conditions, HDL particles undergo extensive remodeling: they lose protective enzymes such as PON1 and acquire proinflammatory proteins including SAA and MPO (35, 78). This transformation converts HDL from an anti-inflammatory, cholesterol-scavenging particle into a dysfunctional—and even proinflammatory—entity, thereby exacerbating cardiovascular risk.

Thus, the SIRT1/STAT3 axis provides a unifying molecular framework that explains the increased CVD risk in RA despite normal or low LDL-C levels. It highlights the central role of STAT3 in generating atherogenic lipoproteins and the contribution of SIRT1 suppression to impaired cholesterol clearance and HDL dysfunction.

### 5.2 Local lipid metabolism and mediator balance

In the rheumatoid joint, the SIRT1/STAT3 axis drives pathological remodeling of the local lipid milieu that directly sustains synovitis. STAT3 activation in FLS and macrophages induces overexpression of key enzymes in the AA cascade, including cyclooxygenase-2 (COX-2) and 5-lipoxygenase (5-LOX), thereby promoting excessive production of proinflammatory eicosanoids such as PGE2 and LTB4 (100, 101). These lipids are central mediators of pain, vasodilation, and leukocyte recruitment. Conversely, SIRT1 deficiency impairs the generation of endogenous proresolving SPMs. SIRT1 normally facilitates the biosynthesis of SPMs; its suppression reduces the expression and activity of 15-lipoxygenase (15-LOX), which converts AA into proresolution lipoxins and mediates the biosynthesis of resolvins from omega-3 fatty acids (102, 103). Consequently, the lipid mediator imbalance in RA extends beyond eicosanoids. Lipidomics studies have revealed significant alterations in other bioactive lipid classes, including oxidized phospholipids (OxPLs) and sphingolipids. Elevated OxPLs act as DAMPs that perpetuate inflammation, while altered sphingolipid metabolism (e.g., increased ceramides) contributes to cellular dysfunction and apoptosis resistance in the synovium (104, 105). Thus, imbalance of the SIRT1/STAT3 axis creates a local lipid mediator “switch” frozen in the “on” position for inflammation and the “off” for resolution, while broader lipidomic alterations further amplify the pathogenic state.

### 5.3 Linking lipid dysregulation back to inflammation

The most deleterious effect of SIRT1/STAT3 axis dysregulation in lipid metabolism is the creation of a self-reinforcing feedback mechanism; the lipid abnormalities it induces further amplify its own dysregulation. Investigators have revealed that saturated free fatty acids (SFFAs) (palmitate, for example), released via enhanced lipolysis, can function as ligands that block SIRT1 activity and, while simultaneously activating proinflammatory signaling pathways, thereby further repressing SIRT1 and activating STAT3 (106). In parallel, ox-LDL, a product of the STAT3-favored milieu, is internalized by macrophages through scavenger receptors, leading to foam cell formation and the release of additional cytokines, including TNF-α, which reinforce STAT3 activation (107, 108).

This circuit establishes a vicious cycle of immune-inflammation–lipid metabolism dysfunction, providing a mechanism basis for RA progression. **Table 1** summarizes the core role of the SIRT1/STAT3 axis in regulating lipid dysregulation in RA.

**Table 1:** Core mechanisms of lipid dysregulation governed by the SIRT1/STAT3 axis in RA.

Level of action	Key mechanisms	Metabolic consequences	Pathophysiological impact	References
Systemic regulation	IL-6/STAT3 drives proatherogenic sdLDL generation, while SIRT1 deficiency impairs LXR-mediated cholesterol efflux (via ABCA1/G1).	Increased oxidized sdLDL and reduced reverse cholesterol transport	Explains the “lipid paradox”, characterized by high CVD risk despite normal/low LDL-C levels.	(52, 76, 77, 98, 99)
Local joint environment	STAT3 induces COX-2/5-LOX, boosting proinflammatory lipids (PGE2, LTB4), whereas SIRT1 loss disrupts production of proresolving SPMs (e.g., lipoxins).	Lipid mediator imbalance, with inflammation persisting and resolution failing, alongside metabolic reprogramming of immune cells.	Directly drives persistent synovitis and chronic pain.	(100–103)
Self-reinforcing feedback loop	SFFAs (e.g., palmitate) inhibit SIRT1 and activate STAT3, while STAT3-generated ox-LDL fuels foam cell formation and releases TNF-α.	Lipid disturbances and inflammation mutually amplify one another.	Establishes a vicious cycle that perpetuates RA progression and elevates CVD risk.	(106–108)

CVD, cardiovascular disease; LDL-C, low-density lipoprotein cholesterol; sdLDL, small dense low-density lipoprotein; LXR, liver X receptor; ABCA1/G1, ATP-binding cassette transporter A1/G1; COX-2, cyclooxygenase-2; 5-LOX, 5-lipoxygenase; PGE2, prostaglandin E2; LTB4, leukotriene B4; SPMs, specialized proresolving mediators; SFFAs, saturated free fatty acids; ox-LDL, oxidized low-density lipoprotein; TNF-α, tumor necrosis factor alpha.

## 6 Targeting the SIRT1/STAT3 axis: therapeutic prospects in RA

### 6.1 SIRT1 agonists: restoring metabolic homeostasis and anti-inflammatory tone

The pharmacological activation of SIRT1 is an attractive strategy to counter its reported suppression in RA. Natural polyphenols such as resveratrol are well-established activators of SIRT1 and have demonstrated efficacy in animal models of arthritis by reducing synovitis, cartilage degeneration, and proinflammatory cytokine secretion (109, 110). Of more relevance, resveratrol has shown antihyperlipidemic activity in adjuvant-induced arthritis rats, regulating the HDL/LDL ratio and preventing oxidative stress through SIRT1-mediated activation of LXR and AMPK cascades (111). The low bioavailability of resveratrol has prompted the development of more effective and selective synthetic sirtuin-activating compounds (STACs), e.g., SRT2104. *In vivo* and *in vitro* studies have demonstrated that, in addition to reducing joint inflammation, SRT2104 reverses high-fat diet (HFD)-induced metabolic defects, supporting dual anti-inflammatory and metabolic effects (112, 113). Correspondingly, in RA, upregulation of SIRT1 is expected to deacetylate and deactivate STAT3, thereby repressing Th17 differentiation, inducing Treg expansion, and promoting macrophage polarization from M1 to M2. As a result, dual activation of PGC-1α and LXR by SIRT1 agonists would promote mitochondrial activity and cholesterol efflux, thereby directly ameliorating the metabolic disturbances of RA. While these preclinical results are promising, clinical translation remains at an early stage. To date, no large-scale randomized controlled trials have evaluated SIRT1 activators specifically in RA patients. The safety and efficacy of compounds such as SRT2104 have been tested primarily in healthy elderly volunteers or in other disease contexts, with no dedicated RA trials reported.

### 6.2 STAT3 inhibitors: breaking the cycle of inflammation and prometabolic signaling

Another rational strategy is direct targeting of the overactive STAT3 node, for example, through inhibition of JAKs, which activate STAT3. JAK inhibitors (e.g., tofacitinib, baricitinib) are already in clinical use for RA, and their success supports the significance of the JAK–STAT pathway (114, 115). Although their activity extends beyond lymphocytes, JAKs are also known to enhance endothelial and metabolic function by partially disentangling inflammation from metabolic risk (116). However, broader inhibition of JAKs is nonspecific to STAT3. More direct approaches include STAT3-specific inhibitors that disrupt SH2 domain-mediated oligomerization (e.g., Stattic/C188-9) or decoy oligonucleotides that compete for the DNA-binding site (117, 118). Although clinical development remains limited, preclinical data are encouraging. Li et al. validated *in vitro* findings showing that Stattic inhibits osteoclast generation by modulating RANKL-triggered STAT3 and NF-κB pathways, representing a novel therapeutic approach for RA (119). Despite this promise, the development of selective STAT3 inhibitors faces major challenges. Given STAT3’s pleiotropic roles in maintaining homeostasis across multiple tissues (e.g., liver, heart, and immune system), systemic inhibition carries a high risk of on-target toxicities, including immunosuppression and metabolic disturbances. Achieving tissue-specific delivery—for instance, to the inflamed synovium—remains a critical hurdle for preserving efficacy while avoiding systemic side effects. Importantly, blockade of STAT3 is expected to downregulate both the proglycolytic and prolipogenic reprogramming of immune cells and FLS, as well as proinflammatory eicosanoid production through inhibition of COX-2 and 5-LOX expression.

### 6.3 Dietary and adjunctive interventions: metabolic priming of the SIRT1/STAT3 axis

Nondrug therapies also represent an alternative approach to modulating the SIRT1/STAT3 axis through upstream regulators of its activity. Caloric restriction (CR) and intermittent fasting are powerful metabolic stimuli that upregulate SIRT1 expression and activation, mediated by increased cellular NAD^+^ levels (120, 121). Although long-term CR is not an acceptable therapy in RA, CR-mimetic diets or NAD^+^-activating molecules (e.g., nicotinamide riboside) may offer a valuable strategy to enhance SIRT1 tone and strengthen its inhibitory control over STAT3 (122). The most immediate nutritional intervention has been supplementation with *n*-3 PUFAs, e.g., EPA and DHA. *n*-3 PUFAs exert diverse actions converging on the SIRT1/STAT3 pathway. They serve as precursors for SPMs (e.g., PGD2, 5-HEP, LXB4), whose reduced production is a characteristic feature of RA. These SPMs play a critical role in inflammation resolution (123, 124). In addition, clinical studies and meta-analyses have demonstrated that *n*-3 PUFA supplementation in RA patients is associated with modest clinical benefits and reduced NSAID use, effects that may be partly mediated by reversal of the SIRT1/STAT3 axis and restoration of the lipid mediator profile (125). **Table 2** highlights the emerging therapeutic perspective of targeting the SIRT1/STAT3 pathway within the metabolic–immune integration of RA. Furthermore, to contextualize the therapeutic potential of SIRT1/STAT3 axis modulation in the current RA treatment landscape, **Table 3** presents a comparative overview illustrating how these strategies differ mechanistically and clinically from established RA therapies.

**Table 2:** Therapeutic strategies targeting the SIRT1/STAT3 axis in rheumatoid arthritis.

Therapeutic strategy	Representative agents	Core mechanisms	Key outcomes	References
SIRT1 agonists	Resveratrol and SRT2104	Activates SIRT1 and inhibits STAT3, promotes anti-inflammatory responses (Treg, M2), and enhances cholesterol efflux (LXR) as well as mitochondrial function.	Dual anti-inflammatory and metabolic benefits	(109–113)
STAT3 inhibitors	Tofacitinib and Stattic	Blocks STAT3 signaling (via JAK inhibition or direct binding), reduces proinflammatory factors, and limits metabolic reprogramming.	Directly interrupts the core inflammatory and metabolic drive	(114–117, 119)
Dietary and adjunctive interventions	*n*-3 PUFAs (EPA/DHA) and NAD^+^ boosters	*n*-3 PUFAs serve as precursors for SPMs, NAD^+^ boosters activate SIRT1, and together these mechanisms promote the resolution of inflammation.	Safe and physiological priming	(120–125)

Treg, regulatory T cell; M2, alternatively activated macrophage; LXR, liver X receptor; JAK, Janus kinase; n-3 PUFAs, omega-3 polyunsaturated fatty acids; EPA, eicosapentaenoic acid; DHA, docosahexaenoic acid; SPMs, specialized proresolving mediators; NAD^+^, nicotinamide adenine dinucleotide.

**Table 3:** Mechanistic and clinical distinctions between SIRT1/STAT3-targeted strategies and existing RA therapies.

Feature	Existing RA therapies (e.g., csDMARDs, bDMARDs, and tsDMARDs)	SIRT1/STAT3 axis-targeted strategies (proposed)	References
Primary mechanism	Immunosuppression/Cytokine neutralization: inhibit lymphocyte proliferation (MTX), neutralize specific cytokines (anti-TNF, anti-IL-6R), or block downstream JAK signaling (JAKi).	Immunometabolic programreprogramming: aims to restore cellular homeostasis by correcting the underlying metabolic and inflammatory imbalance.	(7, 8, 114, 115)
Effect on lipid metabolism	Variable/Indirect: TNFi can transiently alter lipid levels without clear functional improvement of HDL, whereas JAKi effects on lipids are complex and remain under ongoing study.	Direct and dual: aims to simultaneously dampen inflammation and directly enhance atheroprotective pathways (e.g., RCT via SIRT1/LXR, reduction of sdLDL via STAT3 inhibition).	(15, 52, 77, 116, 125)
Target specificity	High (bDMARDs) to broad (JAKi): targets a single cytokine or a family of kinases involved in multiple pathways.	Node-specific: targets a central signaling node (SIRT1/STAT3) that integrates multiple upstream signals, aiming for a more coordinated and physiological reset.	(22, 23, 70, 71)
Therapeutic goal	Symptom control and halt joint damage: primarily focused on reducing synovitis and preventing radiographic progression.	Immunometabolic remission: aims not only for clinical remission but also for resetting the systemic immunometabolic setpoint to reduce comorbid CVD risk.	(9–11)
Potential advantages	Proven efficacy with established safety profiles.	Addresses unmet need: has the potential to treat patients refractory to current therapies and may directly mitigate cardiovascular comorbidities.	(110, 111, 113, 125)
Key translational hurdles	Established: cost, immunogenicity, and infection risk.	Emerging: tissue-specific delivery, long-term safety of metabolic manipulation, and defining clinical trial endpoints that capture dual benefits.	(19, 114)
Clinical status	Standard of care: widely approved and used.	Preclinical/Early clinical: requires validation in RA-specific trials (e.g., SIRT1 activators such as SRT2104 or next-gen STAT3 inhibitors).	(113, 114, 117, 119)

csDMARD, conventional synthetic disease-modifying antirheumatic drug; bDMARD, biologic DMARD; tsDMARD, targeted synthetic DMARD; MTX, methotrexate; TNFi, tumor necrosis factor inhibitor; JAKi, Janus kinase inhibitor; RCT, reverse cholesterol transport; CVD, cardiovascular disease; HDL, high-density lipoprotein; sdLDL, small dense low-density lipoprotein.

## 7 Discussion

The complex pathophysiology of RA has historically been framed through dissociated viewpoints of autoimmune dysregulation and comorbid metabolic syndrome. A paradigm shift is now emerging that views these phenomena as interconnected entities within a broader “immunometabolic” system. In this review, we converge and synthesize persuasive evidence positioning the SIRT1/STAT3 axis as a master control point of this network—an essential sensing and signaling integration node between inflammatory stimuli and metabolic stressors—driving RA pathogenesis and systemic sequelae.

In our view, the antagonism between SIRT1 and STAT3 functions as a rheostat of cellular homeostasis. Under homeostatic conditions, sufficient NAD^+^ concentrations sustain SIRT1 activity, which tonically represses STAT3 through deacetylation to limit inflammation and support catabolic balance. In RA, chronic long-term cytokine stimulation (e.g., IL-6 and TNF-α) and oxidative stress disrupt this delicate balance. This mechanism establishes a pathogenic positive feedback loop between inflammation and metabolic stress, reducing SIRT1 activity and increasing STAT3 acetylation/activation, thereby amplifying inflammation and lipid dysregulation. Such a model helps to reconcile the myriad of clinical hallmarks of RA, including the Th17/Treg imbalance, the activated and invasive phenotype of FLS, and the enigmatic lipid paradox. The axis is not merely associated with these attributes; it actively regulates them, as demonstrated by preclinical experiments in which direct manipulation of SIRT1 or STAT3 produced phenotypic changes in the disease.

The SIRT1/STAT3 axis does not operate in isolation but engages in extensive crosstalk with other key pathways involved in RA pathogenesis. For instance, hypoxia-inducible factor-1α (HIF-1α) is a master regulator of metabolic adaptation to low oxygen, and its activity is closely linked to STAT3-driven glycolysis (126). STAT3 can directly enhance HIF-1α transcription, whereas SIRT1 can deacetylate and destabilize HIF-1α, adding another layer of antagonism. Similarly, the NLRP3 inflammasome—a critical mediator of IL-1β and IL-18 maturation—is modulated by this axis (127). SIRT1 activation suppresses NLRP3 inflammasome activation, whereas STAT3 promotes the expression of NLRP3 components. These interconnections suggest that the SIRT1/STAT3 axis functions as part of a larger, highly integrated regulatory network in which acetylation status and metabolic cues govern multiple downstream pathogenic mechanisms.

The clinical relevance of this model is considerable. Interventions directed at the SIRT1/STAT3 axis do not aim to inhibit immunity or lipid metabolism but rather to restore the normal dialogue between these two systems. Treatment strategies may include pharmacological approaches using the newer generation of SIRT1 activators or selective STAT3 inhibitors, which hold promise for advancing precision, pathophysiology-based therapy. Combination therapy—potentially guided by biomarkers of axis perturbation (e.g., the ac-STAT3/SIRT1 ratio in peripheral blood mononuclear cells [PBMCs])—represents a step toward individualized medicine in RA. Such approaches may enable synergistic effectiveness with reduced drug doses and fewer side effects, while the axis itself provides a mechanistic explanation for the positive impact of nondrug-related measures. For example, the regulatory role of *n*-3 PUFAs can be reframed: beyond supplying precursors for SPMs, they directly influence this axis by enhancing SIRT1 function and abrogating STAT3 signaling.

Nonetheless, several questions and caveats remain. First, what are the temporal dynamics of axis dysregulation in RA? Do imbalances precede and predispose to clinical onset, or do they simply reflect preexisting inflammation? Addressing this requires longitudinal studies in preclinical RA cohorts. Second, as elaborated below, the cell-type-specific effects of globally targeting the SIRT1/STAT3 axis necessitate the development of tissue-specific delivery strategies to maximize synovial efficacy while minimizing systemic toxicity. Third, additional pathways in RA pathogenesis associated with the SIRT1/STAT3 axis warrant investigation, including the HIF-1α pathway and the NLRP3 inflammasome. Exploring these interactions will further unravel the crosstalk among different pathways in RA. Notably, these pathways are also acetylation-dependent and metabolic state-dependent, implying a more global, interconnected system of regulation.

Despite the compelling preclinical rationale for targeting the SIRT1/STAT3 axis in RA, substantial translational hurdles must be addressed before its clinical potential can be realized.

### 7.1 Tissue specificity and targeted delivery

The pleiotropic nature of SIRT1 and STAT3 presents a major challenge. While inhibiting STAT3 in immune cells or FLS is therapeutically desirable, systemic STAT3 blockade could disrupt critical homeostatic functions in other tissues, including hepatocyte regeneration, cardiac protection, and intestinal epithelial barrier integrity, leading to on-target toxicities. Therefore, the development of tissue-specific delivery systems is paramount.

### 7.2 Long-term safety and immunometabolic setpoint

Resetting the immunometabolic setpoint requires long-term modulation of the SIRT1/STAT3 axis. The safety of chronic SIRT1 activation or STAT3 inhibition remains incompletely established. For STAT3 inhibitors, long-term immunosuppression is a primary concern, with potential risks of opportunistic infections and impaired tumor surveillance. For SIRT1 activators, the long-term consequences of sustained deacetylase activity on global cellular function and epigenetic regulation warrant careful evaluation in longitudinal studies.

### 7.3 Regulatory hurdles and clinical trial design

Given that SIRT1/STAT3 axis modulators are designed to impact both inflammatory and metabolic endpoints, their clinical development faces unique regulatory challenges. Clinical trial design will need to evolve beyond standard ACR20/50/70 response criteria. Future trials must incorporate robust, prespecified cardiovascular and metabolic endpoints (e.g., changes in HDL efflux capacity, sdLDL particle number) alongside traditional measures of arthritis activity. Securing regulatory approval will likely require demonstrating a significant impact on these comorbidities to justify a new class of therapy beyond existing disease-modifying antirheumatic drugs (DMARDs).

As a final point, this immunometabolic framework should broaden and reformulate our understanding of RA comorbidity. The markedly increased risk of CVD and metabolic syndrome in RA should be recognized not as coincidental but as a result of SIRT1/STAT3 axis dysregulation that also drives synovitis. This unified concept calls for a comprehensive therapeutic goal: successful RA therapy should not only induce clinical remission but must also reset the immunometabolic setpoint to mitigate systemic comorbidity risk. Future clinical trials of axis-targeted interventions will therefore need to incorporate stringent metabolic and cardiovascular end points in addition to conventional arthritis assessments.

Overall, considering RA in light of the SIRT1/STAT3 axis provides a dynamic, cross-bridging perspective on the immune, inflammatory, and metabolic facets of RA. This perspective challenges the therapeutic silo paradigm and opens promising opportunities for new drug combinations that address not only synovial inflammation but also lipid abnormalities and cardiovascular risk. At present, our target—although still “symptomatic” control—is approaching the ideal of achieving long-term immunometabolic remission in RA.
